# The smile is stronger than the handshake

**DOI:** 10.15694/mep.2020.000068.1

**Published:** 2020-04-13

**Authors:** Lauren Fine, Vijay Rajput

**Affiliations:** 1 Nova Southeastern University

**Keywords:** Handshake, Handwashing, Nonverbal communication

## Abstract

This article was migrated. The article was marked as recommended.

Healthcare providers struggle with the timing of handwashing and the handshake during the visit with patients, we question whether the handshake is even a necessary component of the modern introduction. The Physician-Patient relationship is strongest when built on trust, and typically begins with a traditional handshake. The importance of hand hygiene has become an integral part of the patient encounter. Taking a break for hand-hygiene interrupts the natural flow of the initial introduction and non-verbal body language to our patients. In current time constrained visits, we focus on handwashing instead of on continuous eye-contact with our patient. It is arguable that the elimination of the handshake may allow one to focus on more culturally acceptable universal verbal and non-verbal communication skills that help us to build essential trust with our patients.

## Introduction

Our first-year students struggled with the execution of the right sequence of the initial verbal introduction of themselves, washing their hands and shaking hands with their standardized patients. It comes as a surprise to us that although these steps in communication are practiced by preceptors, students continue to struggle with the awkwardness of the sequence of these formative skills.

It is not that health professionals are incapable of introducing themselves, but rather that the rituals that are considered “required” in our western culture can disrupt the flow of what is usually considered a natural process. It is well-known that hand-washing stewardship reduces hospital-acquired infections and therefore hand-washing must be an integral part of every patient visit ( Pittet and Boyce, 2001). The timing of handwashing not only helps reduce rates of transmitting infection but also allows the culture of greetings with their patients to foster trust.

## Complicated Sequences of Handshake and Handwashing in the Patient Visit

The relationship between physicians and their patients begins upon entry of the physician into the patient’s room. Healthcare providers are taught to knock on the door and enter the patient room. After entering they should both sanitize their hands with hand-sanitizer or by handwashing, and introduce themselves to the patient including their name, role and intent. Patients expect their physicians to wash their hands and are often reassured by being able to see their providers wash their hands. A provider who chooses to wash their hands must negotiate one of three different options. One option is to wash their hands during their introduction, which requires them to turn their back to the patient and talk to the patient at the same time. The second option is to introduce themselves to the patient prior to washing their hands while forgoing the handshake in order to reduce transmission of infection. The third option is to enter the room but ignore the patient as they wash their hands and then introduce themselves with a handshake after washing their hands. All three of these options allow for hand-washing to interrupt the initial greeting to the patient in some way that can be seem unnatural or awkward in our western society.

Use of hand-sanitizer avoids the first of the above scenarios but adds the awkward component of rubbing the hands to dry the hand-sanitizer either in silence or while trying to introduce themselves, with or without eye contact. If a patient offers their hand before the provider’s hands are dry, the provider must negotiate whether they should decline the handshake out of embarrassment of having wet hands or shake hands with the patient and apologize for having wet hands.

After the health care provider shakes the patient’s hand they typically do not re-sanitize, allowing for transfer of any infection the patient is carrying to other objects in the room and on the physician including the physician’s pen, computer and other note-taking tools which will be carried from room to room and may be used by other healthcare providers. Furthermore, even the sink that is used to wash hands may actually contribute to infection and outbreaks ( Lowe *et al.*, 2012). Therefore, while hand-washing does reduce the transmission of infection from physician to patient, it not necessarily reduce transmission of infection from patient to physician and back to other patients and healthcare providers ( Given, 1929). It could be argued that patients should be educated on and held responsible for hand-hygiene as well since doing so reduces rates of infection at least in outpatient settings ( Haverstick *et al.*, 2017).

## Trust and non-verbal communication

Trust is fundamental for future negotiations, adherence and outcome of patient diseases. Interrupting or not having the opportunity to focus on non-verbal communication due to handwashing may have long-lasting and deleterious effects on the ability to build trust in the initial encounter. We teach our learners the importance of non-verbal communications such as a firm handshake, eye-contact, sitting at the level of the patient, anticipatory silence, nodding and appropriate facial expressions in the patient visit. These non-verbal communication nuances have been shown to improve patient satisfaction and build trust in a range of patient encounter types. The smile has been proven through fMRI to release dopamine and more often than not is reciprocated reflexively ( Beamish *et al.*, 2019).

## History and Current Status of Handshaking in the Health Care Profession

The handshake is thought to have originated in ancient times as a way of demonstrating trust and revealing that no weapons are hidden ( Given, 1929). It remains a way of demonstrating respect upon initial introduction to another individual and is often accompanied by other non-verbal forms of communicating engagement, acceptance and gratitude such as eye-contact and a smile.

Hand-shaking may not be necessary for patients to feel satisfied with their provider ( Griffith *et al.*, 2003). In fact, in one study only about 50% of patients reported wanting their physician to shake their hand in the first encounter ( Limon *et al.*, 2016). The importance of non-verbal communications such as smile and direct eye contact far outweighed that of the handshake for both families and health care providers in a NICU with a handshake-free policy ( Parga *et al.*, 2017).

In contrast to the introductory handshake, the handshake at the end of an encounter may carry a deeper meaning and has been shown to reflect patient satisfaction ( Jenkins, 2007). Improved patient-physician interactions improves health outcomes in those patients ( Griffin *et al.*, 2004). Therefore, non-verbal communication, an integral part of the physician-patient communication and relationship, has the potential to influence health outcomes. While one must be cognizant of the fact that a handshake is forbidden in some cultures and orthodox religions and that a handshake may not be universally mandatory, a handshake offered by a patient should be accepted and reciprocated in order to help build trust and rapport.

## Should we teach or require our students to shake hands with their patient?

It seems prudent that the handshake is not necessary and may be an interruption in both verbal and non-verbal communication, the effects of which may outweigh any perceived benefit in building trust. When we teach our students how to negotiate the patient interaction there are two components that may need to be realigned. One, the timing of handwashing and second, the timing and necessity of the handshake. Sanitizing hands before or while entering while forgoing the handshake would also allow the student to focus on other forms of non-verbal communication such as eye contact, body positioning and posture and smiling. If non-verbal communication without a handshake is confidently and gracefully used, most likely no explanation for the lack of handshake will be needed. However, concerted efforts may be needed to widely educate the general community about the underlying reasons for this change in practice. Handwashing before the physical exam would still be necessary, but by this time rapport will have been made and a pause for handwashing would unlikely be a communication barrier. A handshake may be desired to close the encounter with an expression of gratitude and respect. However, students and physicians should be aware that culturally appropriate alternatives to the handshake in both greeting and closure should be considered.

## Conclusion

The handshake is a ritual used to establish a trusting relationship between patient and provider. It may be not desirable or not needed if other body language is used appropriately. Therefore, rather than using a handshake as the norm, it may be a best practice to use cultural and contextual based introductions to our patients.

## Take Home Messages

• The handshake is a contemporary Western form of establishing and communicating respect and trust.
• Handwashing is an expected component of the clinical encounter.
• Other forms of establishing rapport through nonverbal communication should be taught and reinforced in the medical profession.

## Notes On Contributors

Vijay Rajput. MD, is a professor and Chair in Department of Medical Education at Nova Southeastern University, Dr. Kiran C.Patel College of Allopathic Medicine. His area of interest is in bedside teaching, humanism, professionalism and Innovative curriculum development. https://orcid.org/0000-0001-5203-5342

Lauren Fine, MD is an assistant professor in Medical Education at Nova Southeastern University, with interests in the doctor-patient relationship, ethics and humanities.
