# Supplementary material for: The smile is stronger than the handshake
Source: MedEdPublish (2016). 2020 Apr 13;9:68. [Version 1] doi: 10.15694/mep.2020.000068.1 (PMC10697525; doi:10.15694/mep.2020.000068.1)
Supplement: Peer review history

# Reviewer report: Reviewer response for version 1

**DOI:** 10.21956/mep.20018.r30543

**Published:** 2020-06-10

**Authors:** Susmita Reddy Karri

**Affiliations:** 1 Topiwala national medical college and BYL nair charitable hospital, mumbai. india

This review has been migrated. The reviewer awarded 4 stars out of 5 A very well timed article. Even though hand-shake is traditionally considered to be one of the best/ time-tested ways to create patient rapport, especially in the Western world and in an OSCE setting, I agree with the authors that a strong, friendly and confident body language can be more effective in doing the job. Having worked in 3 different countries- India, UK and now Australia, I have almost never offered handshake (unless the patients themselves do so) and never did it affect my patient rapport.

Reviewer Expertise:

NA

No decision status is available

# Reviewer report: Reviewer response for version 1

**DOI:** 10.21956/mep.20018.r30542

**Published:** 2020-04-16

**Authors:** PATRICIA CURY

**Affiliations:** 1 FACERES

This review has been migrated. The reviewer awarded 4 stars out of 5 This is a very appropriate article in epidemic time! It makes us think about our cultural costumes. In Brazil, doctors not only are used to handshake their patients but also to kiss their faces and their families, sometimes. We should discuss these things better with our students, not only about handwashing protocols. Congratulations!

Reviewer Expertise:

NA

No decision status is available

# Reviewer report: Reviewer response for version 1

**DOI:** 10.21956/mep.20018.r30535

**Published:** 2020-04-15

**Authors:** Balakrishnan Nair

**Affiliations:** 1 Centre for Medical Professional Development and University of Newcastle

This review has been migrated. The reviewer awarded 4 stars out of 5 This is a timely article . Handshake is only one way of developing rapport with the patient. As the authors state , there are other ways of doing this and we can learn from eastern cultures. I often go to the waiting room and greet the patient and accompany them to the consultation room . Then start with a smile and chit chat before getting into the formal consultation . During inpatient rounds this will be different; again a smile and friendly manner will go a long way. Patients are anxious and we need to put them at ease . The other major question is if we have clean hands and patients do not have , then what is the solution ?

Reviewer Expertise:

NA

No decision status is available

# Reviewer report: Reviewer response for version 1

**DOI:** 10.21956/mep.20018.r30540

**Published:** 2020-04-14

**Authors:** Thomas Puthiaparampil

**Affiliations:** 1 UNIVERSITI MALAYSIA SARAWAK

This review has been migrated. The reviewer awarded 4 stars out of 5 It is a timely and well written article. Hand hygiene is important, so also are building good rapport with the patient and gaining their confidence. I think the students should be taught to avoid the robotic rituals and rather be aware of the reasons for doing things the way they should be done. As some of the reviewers mentioned, handshake is rather western, not universal, and it is not acceptable in some cultures. However, a warm smile, eye contact and a pleasant greeting is universally acceptable as an introduction. Greeting the patient and taking history does not need hand washing, but physical contact would definitely need it. I agree with the authors.

Reviewer Expertise:

NA

No decision status is available

# Reviewer report: Reviewer response for version 1

**DOI:** 10.21956/mep.20018.r30538

**Published:** 2020-04-14

**Authors:** Puja Dulloo

**Affiliations:** 1 Pramukhswami Medical College

This review has been migrated. The reviewer awarded 4 stars out of 5 Interesting articleIn our country, India, we do not have any custom of handshaking. The age old tradition of folding hand is considered to be a greeting sign but never observed this behavioral aspect during physician-patient relationship except for verbal greeting by saying “Namastey” or “Adab” or other verbal words as per the local language or custom.During the present COVID-19 pandemic, hand-shaking approach of greeting is the most common means of infection spread. Thus the Indian way of hand folding has been accepted as one of the best way to keep any sort of spreadable infection at bay.Addressing the issue of hand-shake and hand sanitation during this hour of global pandemic was well required. Smile, a strong non-verbal communication skill not only as a part of physician-patient relationship but even for humanity aspect. “A warm smile is the universal language of kindness.” – William Arthur Ward.

Reviewer Expertise:

NA

No decision status is available

# Reviewer report: Reviewer response for version 1

**DOI:** 10.21956/mep.20018.r30537

**Published:** 2020-04-14

**Authors:** P Ravi Shankar

**Affiliations:** 1 American International Medical University

This review has been migrated. The reviewer awarded 4 stars out of 5 This is an interesting article especially in the context of the ongoing COVID-19 pandemic. I have worked both in South Asian and Caribbean medical schools. The importance of handwashing and the handshake is emphasized during the OSCE with the standardized patient. In today’s environment the risk of transmitting infections is widely recognized as a drawback of the handshake. The authors do discuss other culturally appropriate alternatives. Folding of hands and doing Namaskar, and a bow may be other forms of greeting. Washing hands both before and after the physical examination is important. I am not sure in what form the traditional western handshake will survive in a post COVID-19 world. Also, most students wash their hands as a routine procedure and the correct technic of handwashing is not always followed. This is an interesting and well-written article which I enjoyed reading.

Reviewer Expertise:

NA

No decision status is available

# Reviewer report: Reviewer response for version 1

**DOI:** 10.21956/mep.20018.r30539

**Published:** 2020-04-14

**Authors:** Deb Halder

This review has been migrated. The reviewer awarded 4 stars out of 5 the authors need to be offered kudos at the first glance as they introduced a topic relevant at the event of COVID-19 pandemic that teaches some habitual practices as mandatory.This is claimed to have been a personal opinion which has maintained an orderly progression of thought and logic; the topic has been reviewed from multilateral perspective of religion, social as well as clinical aspect.that addresses interest is its heightened insight over the issue of hand washing and hand sanitizing. but few more literature would have been suitable to make this paper more argumentative and persuasive with reliability.

Reviewer Expertise:

NA

No decision status is available

# Reviewer report: Reviewer response for version 1

**DOI:** 10.21956/mep.20018.r30534

**Published:** 2020-04-14

**Authors:** Junichi Tanaka

**Affiliations:** 1 tohoku university

This review has been migrated. The reviewer awarded 5 stars out of 5 It was a very interesting article. In Japan, I don't think there is much of a custom to shake hands. However, in Japan, many doctors perform some kind of physical examination. It also serves the purpose of determining something by physical examination. But more importantly, it's done as a ritual of nonverbal communication. With the current coronavirus epidemic, more doctors are avoiding physical exams. I think we are in a time where we are forced to think about how to empathize and communicate without directly touching the patient. It was a very thought-provoking opinion in that regard.

Reviewer Expertise:

NA

No decision status is available

# Reviewer report: Reviewer response for version 1

**DOI:** 10.21956/mep.20018.r30536

**Published:** 2020-04-13

**Authors:** Subha Ramani

**Affiliations:** 1 Harvard Medical School, Brigham and Women's Hospital

This review has been migrated. The reviewer awarded 4 stars out of 5 I read this perspective with interest and hand washing and sanitization is important regardless of a pandemic context. Clearly, many studies have demonstrated the ease with which bacteria and viruses can be spread from clinician to patient, vice versa and to other patients. Non verbal communication with patients and families are very important in building trust and rapport and a handshake has been one of these behaviors in the Western world which many countries in the non-Western world have also adopted.On the other hand, I have seen handshakes without a smile or any other behaviors that indicate rapport. In these cases, the handshake is cursory and not communicating any empathy. Therefore, I agree that we could start thinking about several strategies to build patient relationships beyond a handshake. The current pandemic may have made this issue more urgent, but lessons learned can be applied when the crisis settles.

Reviewer Expertise:

NA

No decision status is available

# Reviewer report: Reviewer response for version 1

**DOI:** 10.21956/mep.20018.r30541

**Published:** 2020-04-13

**Authors:** Trevor Gibbs

**Affiliations:** 1 AMEE

This review has been migrated. The reviewer awarded 4 stars out of 5 During the time of the Covid-19 pandemic, the issue of handwashing is important. In reviewing this paper however, I tried to read it without the coronavirus looking over my shoulder.I felt that it was a well written paper about a very debatable issue regarding those initial and very important first, and last elements of the patient encounter. I have encountered many times, usually during an OSCE where students were marked down for omitting to wash their hands because their prime motive was to make effective patient contact. It is not that hand hygeine is not important, it is but as this paper discusses, when and how is more important. Ask many students during training and they will frequently tell you that they was their hands because it becomes a expected ritual rather than having discussed it, why it exists, and what effect it has on the early communication with the patient.This paper does not provide the answers, it leaves the reader thinking about an accepted practice and as such I would recommend it to all those involved in communication skills and clinical skill development.

Reviewer Expertise:

NA

No decision status is available

# Reviewer report: Reviewer response for version 1

**DOI:** 10.21956/mep.20018.r30544

**Published:** 2020-04-13

**Authors:** Johnny Lyon Maris

**Affiliations:** 1 Southampton GP Education Unit

This review has been migrated. The reviewer awarded 4 stars out of 5 This is a really well timed article. It reads well but I think it could have gone further. General populations over the last 6 weeks have moved from the 'elbow touch' or 'foot bump' as a greeting to social distancing, where personal physical contact is taboo. We now have verbal and non-verbal greetings to establish rapport and trust with no physical exchange. For the future of infection control this may well be the normal.I find a warm verbal greeting, eye contact and a smile cements the start of a consultation and this is where I consider the future lies. To wash your hands after the previous consultation/contact has ended is good practice, to then shake hands 'contaminates' the physician and for infection control purposes, the doctor should then wash their hands again. As the author states the eye contact and rapport built is interrupted while the second hand washing is carried out.Avoiding the handshake is just good clinical practice, and the western world should learn from our Hindu colleagues and use the namaste greeting.

Reviewer Expertise:

NA

No decision status is available
